# Exploring Methylene Blue and Its Derivatives in Alzheimer's Treatment: A Comprehensive Review of Randomized Control Trials

**DOI:** 10.7759/cureus.46732

**Published:** 2023-10-09

**Authors:** Muhammad Usman Hashmi, Ragda Ahmed, Sulafa Mahmoud, Kholood Ahmed, Noura M Bushra, Areeg Ahmed, Batran Elwadie, Amna Madni, Amel B Saad, Nadir Abdelrahman

**Affiliations:** 1 Geriatrics, College of Human Medicine, Michigan State University, East Lansing, USA; 2 Internal Medicine, White River Health, Batesville, USA; 3 Internal Medicine, Michigan State University, East Lansing, USA; 4 Internal Medicine, College of Human Medicine, Michigan State University, East Lansing, USA; 5 Nephrology, Harlem Hospital Center, Columbia University, New York, USA; 6 Family Medicine, College of Human Medicine, Michigan State University, East Lansing, USA

**Keywords:** primary senile degenerative dementia, alzheimer’s dementia, amyloid-β, white matter, synaptic plasticity, methylene blue, hippocampal fibres, hippocampal ca1, axonal degeneration, alzheimer disease

## Abstract

Methylene blue (MB) and its compounds are investigated for their potential benefits in the management of Alzheimer's disease (AD). AD is a widely seen neuropathological disorder characterized by the gradual decline of cognitive abilities, ultimately leading to the development of severe dementia. It is anticipated that there will be a significant increase in the prevalence of AD due to the aging population. Histopathologically, AD is distinguished by the presence of intracellular tangles of neurofibrillary tissues (NFTs) and extracellular amyloid plaques within the brain. MB is a thiophenazine dye with FDA approval for treating several illnesses. Its ease in crossing the blood-brain barrier and potential therapeutic use in central nervous system diseases have increased interest in its application for treating AD. The literature review includes randomized clinical trials investigating MB's potential benefits in treating AD. The findings of the studies indicate that the administration of MB has demonstrated enhancements in cognitive function, reductions in the accumulation of plaques containing beta-amyloid, improvements in memory and cognitive function in animal subjects, and possesses antioxidant properties that can mitigate oxidative stress and inflammation within the brain. This review evaluates the modern and latest research on the application of MB for treating AD.

## Introduction and background

Alzheimer's disease (AD), the prevailing neurodegenerative disorder, is characterized by a progressive decline in cognitive abilities, ultimately resulting in profound dementia [1]. Over 95% of Alzheimer's cases appear late in life, with elderly age being the most significant risk factor. Apolipoprotein E4 allele, chronic inflammatory conditions, cardiovascular illness, and traumatic brain damage are other risk factors [2-5]. AD impacts approximately 10.7% of individuals aged 65 years and above, representing approximately one out of every nine individuals in this age group. In the United States, the proportion of women diagnosed with AD is approximately 66%. The prevalence of AD and other forms of dementia is approximately two times higher in the older Black American population compared to the older White American population. The prevalence of AD and other dementias among older Hispanics is approximately 1.5 times higher compared to older individuals of White ethnicity. The year 2020 witnessed a notable association between the COVID-19 pandemic and a rise of 17% in mortality rates related to AD and other forms of dementia [6]. With the progressive growth of the aged population in the United States, there is a corresponding rise expected in the incidence of both new and existing instances of AD. It is estimated that approximately one out of every three older individuals may ultimately succumb to Alzheimer's or another form of dementia [7]. The estimated population of individuals in the United States who are affected by Alzheimer's dementia is approximately 6.5 million, and this prevalence tends to rise in correlation with advancing age. In the absence of medical advancements aimed at preventing, decelerating, or treating AD, the aforementioned figure has the potential to escalate to 13.8 million individuals by the year 2060 [8].

In terms of histopathology, the characteristic feature of AD is the identification of neurofibrillary tangles (NFTs) within the cell. These NFTs are composed of hyperphosphorylated tau proteins that are organized as either paired helical filaments (PHFs) or straight filaments (SFs). In addition, there are extracellular amyloid plaques composed of aggregated peptide fragment A derived from amyloid precursor protein (APP) [5]. Advanced AD patients' brains dramatically shrink due to significant cell loss. Nevertheless, recent studies have demonstrated that the onset of cognitive symptoms may be preceded by synaptic connection loss and neuronal structural changes in the brain. Additionally, these changes can commence as early as two decades prior to the onset of the disease [9,10]. Considering tau disease, NFT creation extends to multiple brain areas in a predictable six-phase development [11]. Patients typically do not have cognitive impairment throughout these first two phases.

Methylene blue, commonly referred to as MB, serves as a thiophenazine dye that has found extensive application in many fields, such as bacteriology, redox analysis, and medical treatment as an antiseptic for various ailments. This substance has received FDA approval for treating several illnesses, including psychiatric disorders such as claustrophobia and malaria [12-14]. As a result of MB's ease in crossing the blood-brain barrier, there is increased interest in its therapeutic use for conditions affecting the central nervous system [15-17]. Multiple studies have shown that MB reduces the risk of neurodegenerative disorders, including AD, ischemic damage to the brain, as well as status epilepticus and Leber's optic neuropathy [16,18-20]. MB is a low-molecular-weight dye found to contain certain properties that can potentially be beneficial in the therapeutic management of AD [21]. Numerous research studies on its effects in laboratory and clinical settings have been conducted recently due to increased interest in its potential as a therapy option. The literature review reveals that systematic reviews have been published on various disease-modifying drugs to prevent or treat age-related dementia. However, we could not find a comprehensive review specifically about the effects of MB on AD. This review aims to evaluate the latest research on the application of MB for treating AD.

## Review

Methodology

A thorough and extensive search was carried out to identify relevant research investigating the possible therapeutic advantages of MB in the management of AD. The investigation was conducted using electronic databases, namely PubMed and Scopus. The scope of the search was restricted to research that was published only in the English language. No date limits were imposed on the search. In this review, we employed ChatGPT, a state-of-the-art natural language processing model, to create an initial manuscript. ChatGPT was utilized to craft the overall structure of the paper.

Search strategy

The search terms and the obtained results are summarized in Table 1.

**Table 1:** Search terms for the detailed literature search and the obtained results.

Database	Date of search	Search term	Total number of studies
PubMed	08-06-2023	“(Alzheimer Dementia) OR (Alzheimer Dementias)) OR (Dementia, Alzheimer)) OR (Alzheimer's Disease)) OR (Senile Dementia)) OR (Dementia, Alzheimer Type)) OR (Alzheimer-Type Dementia (ATD))) OR (Alzheimer Type Dementia (ATD))) OR (Dementia, Alzheimer-Type (ATD))) OR (Alzheimer Type Senile Dementia)) OR (Primary Senile Degenerative Dementia)) OR (Alzheimer Sclerosis)) OR (Alzheimer Syndrome)) OR (Alzheimer's Diseases)) OR (Alzheimer Diseases)) OR (Alzheimers Diseases)) OR (Senile Dementia, Alzheimer Type)) OR (Acute Confusional Senile Dementia)) OR (Dementia, Presenile)) OR (Alzheimer Disease, Late Onset)) OR (Alzheimer's Disease, Focal Onset)) OR (Familial Alzheimer Disease (FAD))) OR (Familial Alzheimer Diseases (FAD))) OR (Alzheimer Disease, Early Onset)) OR (Early Onset Alzheimer Disease)) OR (Presenile Alzheimer Dementia)”	213,139
PubMed	08-06-2023	“(Blue, Methylene) OR (Methylthioninium Chloride)) OR (Methylthionine Chloride)) OR (Swiss Blue)) OR (Basic Blue 9)) OR (Methylene Blue N)) OR (Chromosmon)) OR (Urolene Blue)”	25,843
PubMed	08-06-2023	“(Blue, Methylene) OR (Methylthioninium Chloride)) OR (Methylthionine Chloride)) OR (Swiss Blue)) OR (Basic Blue 9)) OR (Methylene Blue N)) OR (Chromosmon)) OR (Urolene Blue)) AND (Alzheimer Dementia) OR (Alzheimer Dementias)) OR (Dementia, Alzheimer)) OR (Alzheimer's Disease)) OR (Senile Dementia)) OR (Dementia, Alzheimer Type)) OR (Alzheimer-Type Dementia (ATD))) OR (Alzheimer Type Dementia (ATD))) OR (Dementia, Alzheimer-Type (ATD))) OR (Alzheimer Type Senile Dementia)) OR (Primary Senile Degenerative Dementia)) OR (Alzheimer Sclerosis)) OR (Alzheimer Syndrome)) OR (Alzheimer's Diseases)) OR (Alzheimer Diseases)) OR (Alzheimers Diseases)) OR (Senile Dementia, Alzheimer Type)) OR (Acute Confusional Senile Dementia)) OR (Dementia, Presenile)) OR (Alzheimer Disease, Late Onset)) OR (Alzheimer's Disease, Focal Onset)) OR (Familial Alzheimer Disease (FAD))) OR (Familial Alzheimer Diseases (FAD))) OR (Alzheimer Disease, Early Onset)) OR (Early Onset Alzheimer Disease)) OR (Presenile Alzheimer Dementia)”	158
Scopus	08-06-2023	"Methylene Blue" OR "Methylthioninium Chloride" OR "MB" OR "Methylene Azure" AND "Alzheimer's Disease" OR "Alzheimer Disease" OR "AD"	1,126

Selection criteria

The research articles included in this literature review consisted of randomized controlled trials (RCTs) in humans that investigated the potential benefits of MB in treating AD. The detailed inclusion criteria are included as follows: (1) RCTs only, (2) studies written in English language only, (3) studies involving elderly individuals aged 60 years or older, (4) studies conducted on patients with Alzheimer's dementia in any setting or any country, and (5) studies using MB as an intervention for the treatment of Alzheimer's dementia. 

Laboratory studies, animal studies, reviews, and case reports were excluded. Furthermore, articles written in languages other than English and those with extraneous information, such as research involving different kinds of dementia, were excluded. The selection of research was conducted by evaluating the title, abstract, and full text of each study. During this process, two independent authors screened the reference lists of the identified articles. After the removal of the duplicate, the articles were screened using titles and abstracts. Subsequently, the full texts were reviewed to find the relevant studies as per the inclusion criteria. This process helped to ensure a thorough and accurate screening process, increasing the reliability of the chosen studies. After this selection process, a total of six RCTs were included in this research.

Quality assessment

**Figure 1:**
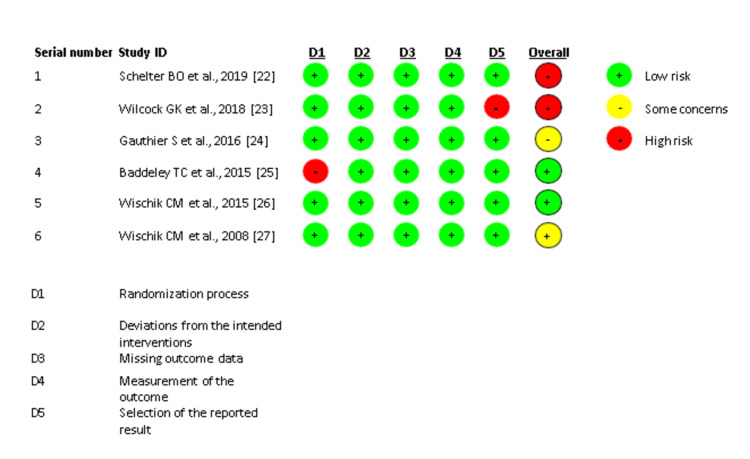
Risk of bias summary.

**Figure 2:**
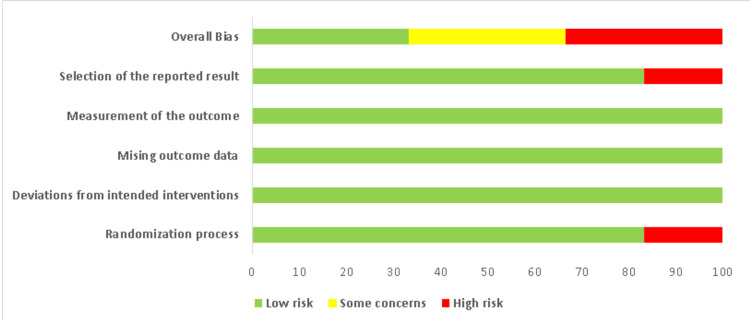
Risk of bias graph.

The quality of the included studies was assessed using the Cochrane method for assessing the risk of bias in RCTs. The studies were divided into three categories: high risk of bias, low risk of bias, and uncertain risk of bias. The risk of bias summary and graph were prepared using the web application Risk-of-bias Visualization. The study summary is provided in Figures 1, 2.

Results

Five out of six included RCTs demonstrated that MB enhanced cognitive performance and decreased beta-amyloid plaque buildup, which is a characteristic feature of AD. The research findings indicated that the administration of MB resulted in enhanced memory and cognitive abilities among those diagnosed with AD. It is believed that inflammatory and oxidative damage in the brain contribute to the progression of AD. Interestingly, the antioxidant capabilities of MB have been shown to mitigate inflammatory and oxidative damage in the brain. The significant results of the included research studies are succinctly presented in Table 2.

**Table 2:** An overview of the key features of the studies that have been included in the evaluation. LMTM: leuco-methylthioninium bis-hydromethanesulfonate; MT: methylthioninium; MTC: methylthioninium chloride; ADAS-cog: Alzheimer’s Disease Assessment Scale-cognitive subscale; ADCS-ADL: Alzheimer’s Disease Co-operative Study-Activities of Daily Living; ADCS-CGIC: Alzheimer’s Disease Cooperative Study-Clinical Global Impression Change; WBV: whole brain volume; LVV: lateral ventricular volume; RCT: randomized controlled trial.

Sr #	Authors	Publication year	Age group	No. of patients	Study duration	Study type	Intervention group, medication's name, dosage, and administration method	Control (placebo) group, medication's name, dosage, and administration method	Outcome measures	Results
1	Schelter et al. [22]	2019	<90 years	1,162	First RCT = 15 months, second RCT = 18 months	Post hoc analysis of two phase III RCTs	Oral LMTM at 150 and 250 mg/day doses in the first RCT and LMTM 200 mg/day in the second RCT	Oral LMTM 8 mg/day	ADAS-cog 11, ADCS-ADL 23 scales, WBV, and LVV after 65 weeks	"Both monotherapy and add-on therapy result in extremely significant differences in cognitive decline and brain atrophy in patients with above-threshold plasma levels of MT. At 8 mg/day as monotherapy or as an adjunct to symptomatic therapies, hydromethylthionine shows pharmacological effects on brain structure and function. A plateau is observed at higher dosages, and higher dosage is consistent with the absence of dose-response observed in the phase III trials. The treatment benefit is expected to be greatest at 16 mg/day as monotherapy."
2	Wilcock et al. [23]	2018	<90 years	795	18 months	RCTs	The recommended dosage for LMTM used orally was 100 mg, to be taken twice a day	The dosage for oral LMTM was 4 mg administered either twice a day or once daily	The patients were seen once every 13 weeks, including the last session for therapy occurring at week 78 and the last safety check occurring at week 82 after they had stopped receiving treatment. These are the scales that were utilized: ADCS-CGIC, ADCS-ADL scale, ADAS-cog, and ADCS-cog	Patients who received LMTM as monotherapy at either of the two doses tested had constantly superior results than patients who got the same doses as an add-on to cholinesterase inhibitors and/or memantine. LMTM may be beneficial as single-agent therapy and 4 mg twice a day may be as effective as greater doses
3	Gauthier et al. [24]	2016	<90 years	891	15 months	RCTs	The prescribed dosage for LMTM was 75 mg administered twice a day as well as 125 mg administered twice daily	PO of 4 mg LMTM twice a day	ADAS-Cog, ADCS-ADL scale	The primary analysis for this study was negative, and the results do not suggest a benefit of LMTM as an add-on treatment for patients with mild-to-moderate AD
4	Baddeley et al. [25]	2015	___	321	24 months	RCTs	The dosages of oral MT administered were 69 mg/day, 138 mg/day, and 228 mg/day	___	ADAS-cog11, change in regional cerebral blood flow	At a minimal dose of 138 mg MT/day, a significant therapeutic benefit was shown on the ADAS-cog scale in moderate patients after 24 weeks and in both mild and moderate Alzheimer’s dementia after 50 weeks. However, at the same time points, the effect of 228 mg MT/day was less than that of 138 mg MT/day. After 20 weeks of therapy, a similar profile of drop in rCBF was observed in mild individuals. As a result, there was no dosage response relative to the nominal dose for any of these outcomes
5	Wischik et al. [26]	2015	N/A	321	Two years	RCTs	Oral MT 69, 138, and 228 mg three times a day	N/A	ADAS-cog11, change in regional cerebral blood flow	"At 24 weeks, there were vital treatment benefits in two distinct populations when the daily dose of 138 mg was used: in moderate subjects on the ADAS-cog scale and two other clinical scales." Both mild and moderate Alzheimer’s dementia showed improvement after 50 weeks of the ongoing treatment as measured by the ADAS-cog scale. Due to dose-dependent dissolution and absorption restrictions, the greatest dose administration was hampered. The daily minimal dose of safe and effective MT is 138 mg, which shows that further research on MT in AD is recommended
6	Wischik et al. [27]	2008	___	332	Over 50 weeks	RCTs	MTC oral at 30, 60, and 100 mg three times a day	Placebo	ADAS-cog	At the 60 mg dose, MTC produced a significant improvement versus the placebo group. The overall effect size was -6.8 ADAS-cog units, compared to a fall of 7.8 units in the placebo group. MTC has shown great efficacy in mild and moderate AD

Discussion

MB is a chemical known as a heterocyclic phenothiazine, which finds application as a colorant. This substance has the ability to readily traverse the BBB. In 1996, in a laboratory setting, it was found that MB can prevent the clumping of tau proteins. It is believed that MB stops tau proteins from binding to each other [28]. The compound hinders the process of tau aggregation, which is a key factor in the formation of tangled neurofibrillary fibers. In cell models and thin sections of brain tissue (that preserved some of the original cytoarchitecture and cellular organization of the intact brain), MB induced autophagy. This suggests a possible mechanism for MB in the reduction of tau protein aggregation within the neuronal tissue [1,3,4,12,29].

It is interesting to note that in clinical trials, MB has demonstrated its ability to target several molecular pathways associated with AD and showed positive effects [30]. Mitochondrial disruption and oxidative stress play essential roles in cellular aging and senescence. MB may help delay mitochondrial dysfunction with aging and decrease complex IV in AD. Furthermore, increasing research indicates that MB has numerous molecular targets involved in the pathogenesis of the AD route [31].

According to a recent study, a subset of patients who were administered leuco-methylthioninium bis-hydromethanesulfonate (LMTM), a modified version of MB, as the sole treatment, exhibited a significant reduction in the extent of illness advancement compared to the control group [23]. Studies have shown that MB slows the progression of AD and other forms of tauopathy. However, due to its hydrophilic nature, MB’s uptake into the brain is restricted. Nanoparticles (NPs) loaded with MB could offer a more effective treatment for Alzheimer’s and related disorders [32].

Another research study stated that macroautophagy is a cellular process that helps maintain normal cellular homeostasis and plays a significant role in protecting against the progression of neurodegenerative diseases. In this study, it was found that MB induces macroautophagy in HT22 hippocampal neuronal cells. To protect neurons from serum deprivation, MB induces macroautophagy via the 5'-adenosine monophosphate-activated protein kinase pathway [33].

Lee et al. conducted a study in which they demonstrated that photoexcited molecules of MB possess the ability to impede the aggregation of Aβ42 in an in vitro setting. They used multiple photochemical analyses. The *Drosophila* AD model used in this in vivo study also demonstrated the potent ability of photoexcited MB to decrease synaptic toxicity. Consequently, the nervous system's neuromuscular junction (NMJ) experienced reduced damage, resulting in improved motor function and a decreased presence of brain vacuoles [34]. The observed inhibition was ascribed to the oxidative reaction of Aβ42 facilitated by singlet oxygen produced during the photoexcitation of MB. In addition, this research has also shown that photoexcited MB might disaggregate pre-existing Aβ42 clumps and lessen the cytotoxic effects. These findings suggest that photodynamic therapy may improve the efficacy of MB in photodynamic therapy for AD in the future. Considering its photosensitive efficacies, we assumed that singlet oxygen generated through MB under light might contribute to both the beneficial and toxic effects of MB. Another study indicated that low-dose LMTM may be efficacious as a single therapeutic agent. This study concluded that 4 mg twice daily and higher doses might decrease brain atrophy [23]. Additionally, the present study reported that the safety characteristics of LMTM remained in line with prior investigations, and no novel safety concerns were detected. The prevailing adverse events seen in this study primarily encompassed gastrointestinal diseases, which exhibited varying degrees of severity ranging from mild to moderate [23].

In a recent study, researchers attempted to improve the bioavailability of MB in the brain by creating novel hydrophobic NPs. These NPs had an average particle size of 136.5±4.4 nm, which is suitable for BBB permeation, and demonstrated sustained drug release for up to 144 hours with no initial burst release. SH-SY5Y neuroblastoma cells are useful research tools in the field of neurobiology. SH-SY5Y cells are associated with the basal endogenous expression of tau proteins. Interestingly, the researchers found that treatment with the MB-NPs significantly decreased the levels of both endogenous and overexpressed tau protein in SHSY-5Y cells and transfected HeLa cells (overexpressing tau protein) [32].

According to another article, in vitro phosphorylation of tau mediated by MARK4 is inhibited by MB. The authors used a cell-free kinase assay method to examine tau phosphorylation by MARK4 in the presence or absence of MB. When MB was introduced directly to the phosphorylation process, they observed a concentration-dependent reduction in tau phosphorylation. This observation suggests that MB directly affects MARK4 kinase activity, reducing tau phosphorylation [31].

Another study explained the mechanism by which MB influences cognition in a transgenic mouse model. It demonstrated that MB prevents AD through a mechanism involving cytochrome c oxidase and mitochondria. MB also enhances critical mitochondrial biochemical pathways and has been shown to delay cellular senescence. Additionally, MB possesses neurometabolic systems that contribute to the strengthening of memory and the protection of neural cells. The administration of MB therapy resulted in enhanced social behavior in transgenic mice, ameliorated cognitive impairments in both spatial and non-spatial memory domains, reduced beta-amyloid deposition in the hippocampus and cortex, normalized alanine and lactate levels, and had positive effects in both curative and preventive contexts [35].

MB is a widely recognized pharmaceutical agent that has been extensively utilized over an extended period of time due to its versatile applications and limited occurrence of adverse effects. Due to its ability to readily traverse the BBB, MB is becoming more and more sought after in biomedicine as a therapy option for conditions affecting the central nervous system. Previous studies implicate MB in both memory enhancement and neuroprotection. The results suggest that MB may benefit from chronic cerebral hypoperfusion conditions, such as mild cognitive impairment, vascular dementia, and Alzheimer's. Tau aggregation pathology is becoming increasingly recognized as an essential substrate of clinical dementia and a therapeutic target [9,19,36].

In the context of AD, it has been observed that clinical worsening occurs concurrently with the formation of aggregated tau protein. The pursuit of inhibiting tau protein aggregation is a promising therapeutic avenue for the modification of AD. The utilization of MB may have potential in the treatment and prevention of this neurodegenerative condition [10].

A study by Schelter et al. investigated the effect of hydromethylthionine, a derivative of MB. The study's findings suggest that hydromethylthionine has dose-dependent effects on cognitive decline and brain atrophy in mild-to-moderate AD. The study found that the drug has pharmacological activity on brain structure and function at the 8 mg/day dose as monotherapy or as an add-on to symptomatic treatments. Treatment benefit is maximal at 16 mg/day as monotherapy [22]. These findings contribute to Alzheimer's research by providing insight into the potential efficacy of hydromethylthionine as a treatment for AD. The study also highlights the importance of concentration-dependent dosing when developing new drugs for AD. It suggests that further research is needed to determine the optimal dosing strategy for hydromethylthionine.

Conversely, a review study by Hashweh et al. did not support these findings. As per this study, hydromethylthionine failed to achieve primary efficacy endpoints in terms of slowing disease progression at doses of 150-250 mg daily. This dose range was initially anticipated to be therapeutic, compared to a placebo dose of 8 mg per day. This finding suggests that at these doses, the drug may not be effective in treating AD [37]. This study also notes that the results of the phase III trial by Wilcock et al. could have a confounding bias. Particularly, LMT-X monotherapy versus add-on therapy to AD-approved drugs was found to have statistically significant benefits on cognitive results. The findings of Wilcock et al. are likely to be confounded by the fact that those who were on cholinesterase inhibitors or memantine may have had a worse disease prognosis and might have declined faster regardless of any impact of the experimental drug. It is important to note that the most current pharmacokinetic study of LMT plasma levels that considered clinical and imaging information refuted this claim of the therapeutic benefits of MB against AD. Additionally, TauRx Therapeutics (Aberdeen, United Kingdom), the drug's manufacturer, also played a key role in research planning, execution, data analysis, and report preparation in all trials employing hydromethylthionine. This practice may be debatable due to financing (or sponsorship) bias. Therefore, it is suggested that in order to investigate the dose and other aspects of hydromethylthionine trials in humans, it is vitally necessary to better characterize the drug's pharmacological activities. A randomized study design along with maintaining proper blinding is recommended to further explore the therapeutic role of MB in the treatment of AD. Above all, the best way to guarantee objectivity is to keep the funding source apart from the researchers managing the data and conducting the study.

Limitations

It is critical to recognize some limitations of this literature assessment. Firstly, the study selection process was limited to only peer-reviewed articles, which may have excluded relevant studies not published in academic journals or other sources. Secondly, the studies included in this review varied widely in terms of their sample sizes, methodologies, and populations, which makes it difficult to draw firm conclusions about the effectiveness of MB for treating cognitive impairment. Thirdly, while the studies reviewed here suggest that MB may have potential as a cognitive enhancer, many of these studies were conducted on small groups, which may limit generalizability to more extensive and diverse populations. In addition, there was the issue of publication bias and potential conflicts of interest among researchers. Hence, more research is warranted to understand the effects of MB on larger, more diverse groups, especially people suffering from cognitive impairments or dementia.

## Conclusions

While MB shows promise as a treatment for neurological disorders, it has some limitations and challenges that must be addressed. MB is a promising treatment for neurological disorders, including Alzheimer's, Parkinson's, and traumatic brain injury. Animal studies and clinical trials have demonstrated its potential to improve cognitive function, reduce oxidative stress, and protect against neurodegeneration. However, some studies have found adverse effects and safety concerns with MB. Existing studies have limitations such as small sample sizes, short treatment durations, and dosage and administration route differences. While MB shows promise as a treatment for neurological disorders, further research is needed to determine its safety and efficacy. Large-scale, well-designed clinical trials are required to confirm the beneficial effects of MB and determine the optimal dosages and administration routes. More research is also required to understand the mechanisms underlying its therapeutic and potentially harmful effects.
